# Intravesical Interferon Therapy vs Hyaluronic Acid for Pain Among Female Individuals With Interstitial Cystitis: A Randomized Clinical Trial

**DOI:** 10.1001/jamanetworkopen.2024.4880

**Published:** 2024-04-08

**Authors:** Si-hong Shen, Liao Peng, Xiao Zeng, Jie Zhang, Hong Shen, De-yi Luo

**Affiliations:** 1 Institute of Urology, Department of Urology, West China Hospital, Sichuan University, Chengdu, Sichuan, China; 2 Pelvic Floor Diseases Center, West China Tianfu Hospital, Sichuan University, Chengdu, Sichuan, China

## Abstract

**Importance:**

Interstitial cystitis (IC) is a debilitating condition. Although viral infection is a potential etiological cause, few studies have detected the effect of antiviral treatment.

**Objective:**

To determine the efficacy and safety of intravesical interferon instillation compared with hyaluronic acid in female patients with IC.

**Design, Setting, and Participants:**

This double-masked, randomized phase 2/3 clinical trial with parallel group design was implemented from October 2022 to April 2023 and had a 6-month follow-up period. The study was conducted at a single center. Eligible participants were female patients aged 18 to 70 years with a diagnosis of IC for more than 6 months. The last visit took place in October 2023. Data were analyzed between October and November 2023.

**Intervention:**

Patients were randomized 1:1 to receive either intravesical instillation of interferon or hyaluronic acid.

**Main Outcomes and Measures:**

The primary end point was change in visual analog scale pain score. Secondary end points included changes in voiding frequency, functional bladder capacity, symptom index, and global response assessment. Adverse events were closely monitored.

**Results:**

Among the 52 patients, the mean (SD) age was 50.0 (14.1) years and they were randomized to either the interferon group (26 [50%]) or hyaluronic acid (26 [50%]). The visual analog pain score showed the interferon group decreased more significantly than hyaluronic acid (−1.3; 95% CI, −2.3 to −0.3; *P* = .02) at month 6, with 20 patients (77%) exhibiting a 30% or higher reduction in pain compared with baseline. Secondary end points of voiding frequency, functional bladder capacity, and nocturia episodes showed no significant difference between 2 therapies. However, interferon showed a significantly higher reduction in the Interstitial Cystitis Symptom Index (−3.0; 95% CI, −5.3 to −0.7; *P* = .01) and the Problem Index (−2.5; 95% CI, −4.5 to −0.4; *P* = .02) at month 6, with 22 patients (85%) presenting as moderately or markedly improved. The frequencies of adverse events were similar between 2 groups. Only 1 patient discontinued hyaluronic acid because of poor effectiveness.

**Conclusions and Relevance:**

In this randomized clinical trial, female patients with IC could benefit from intravesical interferon therapy, without serious adverse events. These results offered hope for antiviral approaches in IC, but larger-scale, multicenter trials and long-term follow-up should be considered.

**Trial Registration:**

ClinicalTrials.gov Identifier: NCT05912946

## Key Points

**Question:**

Does intravesical interferon instillation offer a novel treatment option for female patients with interstitial cystitis (IC)?

**Findings:**

In a randomized clinical trial of 52 patients with IC, intravesical interferon treatment led to a significant reduction in visual analog scale pain score, and Interstitial Cystitis Symptom and Problem Index scores at month 6 compared with hyaluronic acid, demonstrating potential efficacy in IC management.

**Meaning:**

These findings suggest the intravesical interferon instillation holds promise as an effective and well-tolerated therapeutic option for patients with IC, emphasizing the importance of antiviral approaches in improving patient care and quality of life.

## Teaser

This randomized clinical trial examines the safety and efficacy of intravesical interferon instillation compared with hyaluronic acid in female patients with interstitial cystitis.

## Introduction

Interstitial cystitis (IC) is a chronic and debilitating condition that can significantly diminish a patient’s quality of life, with increased urinary frequency, urgency, and pelvic discomfort or pain, particularly during bladder filling, without any other identifiable pathological findings.^1^ The prevalence of IC in the general population varies from 2% to 17.3%, with rates typically higher in female patients.^2^ Despite extensive research, the precise pathogenesis and underlying mechanisms of IC remain elusive, leading to challenges in diagnosis and treatment.^1^ In some cases, approximately 10% of diagnosed patients resort to invasive surgical procedures,^3^ with a 23% risk of failure to improve symptoms,^4^ emphasizing the urgent need for a deeper understanding of the molecular mechanisms.

The possible pathogenesis includes postinfection autoimmune responses, mast cell activation, urothelial dysfunction, neurogenic inflammation, and metabolic disorders.^5,6^ Increasing evidence suggests that Hunner-type IC (HIC) and non-Hunner-type IC (NHIC) are distinct conditions with varying etiologies and pathophysiological mechanism.^7^ Urothelial denudation within IC bladders results in reduced production of crucial cell surface protectants.^6,8^ While intravesical hyaluronic acid (HA) instillation could help improve the defective bladder glycosaminoglycan layer, relapse often occurs upon discontinuation, as most retrieved studies were nonrandomized and had scarce numbers,^9,10,11^ and the long-term follow-up outcomes are limited.^9,10^

It raises the question of whether external factors continue to damage the urinary epithelium, resulting in barrier disruption. Current research suggests that a virus may be the original pathogen leading to the development of IC. Studies have detected a remarkably high positivity rate of BK polyomavirus (BK virus) and JC virus in the urine of patients with IC using single-cell sequencing.^12,13,14^ Meanwhile, it has been reported that intravesical cidofovir treatment was effective in decreasing viral loads of JC virus and BK virus and reducing symptoms.^13^

Numerous studies have reported that interferon, which is involved in the antiviral response, can negatively regulate JC virus and BK virus infection.^15,16^ Studies have demonstrated that interferons could effectively inhibit both early and late transcription as well as viral replication of JC virus.^16^ Additionally, treatment with interferons could negatively regulate the expression of BK virus viral protein, limit viral replication, and regulate the host immune system.^15^ Therefore, interferon may be a potential specific drug for the treatment of IC, with the potential mechanism may involve the inhibition of BK virus and JC virus replication, and the modulation of immune response. We performed a randomized clinical trial to evaluate the benefits of intravesical interferon therapy compared with HA in the treatment of IC in female patients. We hypothesized that intravesical interferon instillation would demonstrate a significant improvement in bladder pain, urinary symptoms, and overall quality of life compared with HA.

## Method

### Study Design and Patient Selection

This randomized clinical trial was a double-masked, 1:1 controlled phase 2/3 trial with parallel group design, which conducted in our center from October 2022 to April 2023. The procedures used in this study were in accordance with the principles of the Declaration of Helsinki of the World Medical Association on human experimentation. Local ethics committee approval was obtained from the Ethics Committee on Biomedical Research at West China Hospital of Sichuan University. An independent data and safety monitoring board oversaw the study’s progress and safety data. This trial follows the Consolidated Standards of Reporting Trials (CONSORT) reporting guideline. The patients in this article gave written informed consent to the publication of their case details. The full trial protocol is provided in Supplement 1.

Study participants were female patients aged 18 to 70 years who were diagnosed with IC in the absence of infection or tumor according to the Canadian Urological Association and American Urological Association Guidelines.^9,10^ The inclusion criteria were (1) having symptoms for more than 6 months, (2) having an O’Leary-Sant Interstitial Cystitis Symptom Index (ICSI) and O’Leary-Sant Interstitial Cystitis Problem Index (ICPI) score 18 or higher, (3) consenting to bladder perfusion therapy, and (4) actively cooperating with the follow-up of researchers. Exclusion criteria included (1) previous history of allergy to interferon or HA drugs; (2) having comorbid conditions, such as serious heart, lung, liver, kidney or blood diseases, liver function abnormalities, kidney insufficiency; (3) female patients who were pregnant or lactating; (4) having urinary tract infection within 2 months; (5) a history of hepatitis B, hepatitis C, or human immunodeficiency disease; (6) a history of bladder hydrodistension, transurethral resection of bladder, and sacral neuromodulation within 3 months; and (7) treatment with oral medications or intravesical instillation within 3 months.

### Randomization and Interventions

The predefined sequence was generated by a statistician who was uninvolved in study enrollment, assessment, or data collection, using computer-generated numbers in a 1:1 ratio. A random assignment number was allocated to each patient and provided to the investigator via telephone to maintain blinding. The investigator responsible for preparing the study medication was not involved in study enrollment, assessment, or data collection. The bladder instillations were indistinguishable, with only the participant number differing on the syringe label. The investigators involved in participant recruitment, treatment, and assessment remained unaware of the participant’s randomization. Throughout the study, participants were kept unaware of their treatment assignment. Bladder instillations were administered using a standardized technique by a physician who was blinded to the treatment details. The interferon group received installation of 1 mL of 300 international units recombinant human interferon alpha-2b injection with 40 mL sterile saline. The HA group received instillation of 40 mg per 50 mL of HA solution. The treatment plan was weekly instillations over a 4-week period, followed by monthly interferon instillations for the subsequent 4 months. Patients were instructed to avoid urinating for at least 30 minutes after each instillation to enhance its bladder retention.

### Outcome Evaluation

Baseline demographics, clinical characteristics, and a voiding diary were collected. Follow-up visits were conducted at 1 month, 3 months, and 6 months after the first instillation at the outpatient department. The primary end point was the change in the visual analog scale (VAS) between the 2 groups at the 1-month, 3-month, and 6-month follow-up assessments compared with the baseline.

VAS is a pain intensity numerical rating scale from 0 indicating no pain to 10 indicating worst ever pain, and a clinically important pain relief was a reduction in pain of approximately 30% from baseline.^17^ Secondary end points were changes in 3-day voiding diary that including 24-hour urinary frequency, times of nocturia, and functional bladder capacity (the maximum voiding volume on a 3-day voiding diary),^18^ ICSI and ICPI questionnaires (included 4 questions assessing the severity of urgency, frequency of urination, nocturia, and level of pelvic pain),^19^ and a symmetric 7-point global response assessment (GRA) scale (included markedly worse, moderately worse, slightly worse, no change, slightly improved, moderately improved and markedly improved) between the 2 groups at the 1-month, 3-month, and 6-month follow-up assessments compared with the baseline. Moderately improved or markedly improved were defined as treatment responder, which is at least a 2-point improvement in GRA.^20^ Adverse events were closely monitored from the time of first infusion through 1 month post last instillation. Urine analysis, blood chemistry, and blood routine were recorded at patient visits.

### Statistical Analysis

The prespecified statistical analysis plan is provided in study protocol. All analyses were performed on the intention-to-treat (ITT) population, and all available participant data at each time point were included in the analyses regardless of treatment adherence. Clinical data with continuous variables are presented as the mean (SD) and categorical variables are presented as No. (%). The baseline characteristics of the treatment groups were compared with the χ^2^ test for discrete variables and the Mann-Whitney test for continuous variables. Categorical variables were analyzed with χ^2^ or Fisher exact tests. The normality of the outcome variables was assessed using the Shapiro-Wilk normality test. For variables that followed a normal distribution, 2-way repeated measures analysis of variance was applied with change from baseline as the dependent variable. Treatment, time, and the treatment × time interaction were independent variables. Otherwise, we used the Scheirer-Ray Hare test as an alternative statistical analysis method. All analyses were performed by SPSS version 24.0 for Windows (IBM), and *P* < .05 was considered statistically significant. Data were analyzed between October and November 2023.

In this study, we used a superiority trial design. We conducted the power and sample size calculation using the PASS calculator.^21,22^ The probability of the VAS score reduction in the interferon group population was approximately 4.5, while the HA group was 2.9 to 3.6. As such, a minimum of 13 patients was required for each group to achieve 90% power at the 2.5% level to detect a 0.7 difference. Based on a 20% dropout rate, a final sample size of 17 participants for each group was required. Additional information is listed in study protocol in Supplement 1.

## Results

### Patient Demographics

Among the 52 patients screened from a total of 67, 26 patients (50%) were assigned to the intravesical interferon alpha-2b group and 26 patients (50%) were assigned to the HA group. There were no statistically significant differences in baseline characteristics and assessment scores between the groups (Table 1). The mean (SD) age of all patients was 50.0 (14.1) years, and the duration of IC and PBS symptoms was 4.1 (2.5) years. A history of intravesical instillation was found in both the interferon alpha-2b and HA groups (11 [42%] vs 15 [58%]), as well the history of oral medication (21 [81%] vs 20 [77%]) in the past (before 3 months). Hunner ulcers were present in both the interferon alpha-2b and HA groups (10 [38%] vs 9 [35%]). In both the interferon alpha-2b and HA groups, the mean (SD) baseline 24-hour voiding frequency (20.0 [5.5] vs 19.7 [4.5]), nocturia episodes (4.1 [2.2] vs 5.0 [3.1] mL), and functional bladder capacity (132.7 [60.3] vs 118.2 [46.2]) were comparable. Additionally, in both the interferon alpha-2b and HA groups, the mean (SD) scores of ICSI (13.9 [1.0] vs 14.9 [1.2]), ICPI (13.5 [1.0] vs 14.0 [1.3]), and VAS (7.8 [1.4] vs 8.2 [1.1]) were generally similar (Table 1).

**Table 1.** Baseline Characteristics in the Intention-to-Treat Population

Characteristics	Patients, No. (%)		
	Interferon alpha-2b (n = 26)	Hyaluronic acid (n = 26)	Total (n = 52)
Age, y			
≤40	5 (19)	7 (27)	NA
>40	21 (81)	19 (73)	NA
Mean (SD)	50.7 (12.9)	49.2 (15.8)	50.0 (14.1)
BMI, mean (SD)	23.8 (4.8)	22.2 (1.2)	23.0 (3.5)
Duration, mean (SD), y	4.0 (2.7)	4.3 (2.4)	4.1 (2.5)
Menopause			
Yes	19 (73)	17 (65)	36 (69)
No	7 (27)	9 (35)	16 (31)
History of oral medication			
Yes	21 (81)	20 (77)	41 (79)
No	5 (19)	6 (23)	11 (21)
Previous intravesical instillation			
Yes	11 (42)	15 (58)	26 (50)
No	15 (58)	11 (42)	26 (50)
Hunner ulcers			
Yes	10 (38)	9 (35)	19 (37)
No	16 (62)	17 (65)	33 (63)
No. of times voiding frequency in 24 hours, mean (SD)	20.0 (5.5)	19.7 (4.5)	19.9 (4.9)
Functional bladder capacity, mean (SD), mL	132.7 (60.3)	118.2 (46.2)	125.5 (53.0)
No. of nocturia episodes, mean (SD)	4.1 (2.2)	5.0 (3.1)	4.6 (2.7)
ICSI score, mean (SD)	13.9 (1.0)	14.9 (1.2)	14.4 (1.2)
ICPI score, mean (SD)	13.5 (1.0)	14.0 (1.3)	13.7 (1.2)
VAS score, mean (SD)	7.8 (1.4)	8.2 (1.1)	8.0 (1.2)

Abbreviations: BMI, body mass index, calculated as weight in kilograms divided by height in meters squared; ICPI, interstitial cystitis problem index; ICSI, interstitial cystitis symptom index; NA, not available; VAS, visual analog scale.

**Figure 1.**
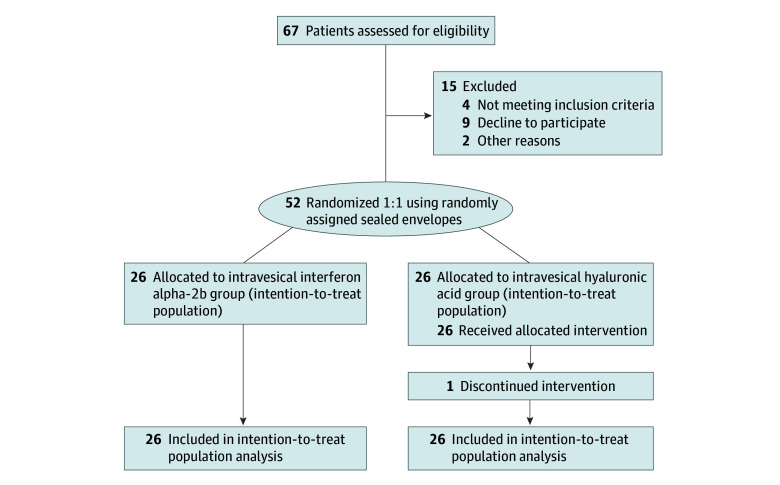
Randomization and Study Population

A total of 51 patients (98%) completed the 8-session of intravesical interferon alpha-2b and HA instillation therapies. One patient discontinued the intravesical HA therapy because of poor effectiveness but remained engaged in all follow-up assessments, and was excluded from the per protocol analysis. Nobody withdrew from follow-up. The randomization and study populations are presented in Figure 1.

### Outcomes

**Figure 2.**
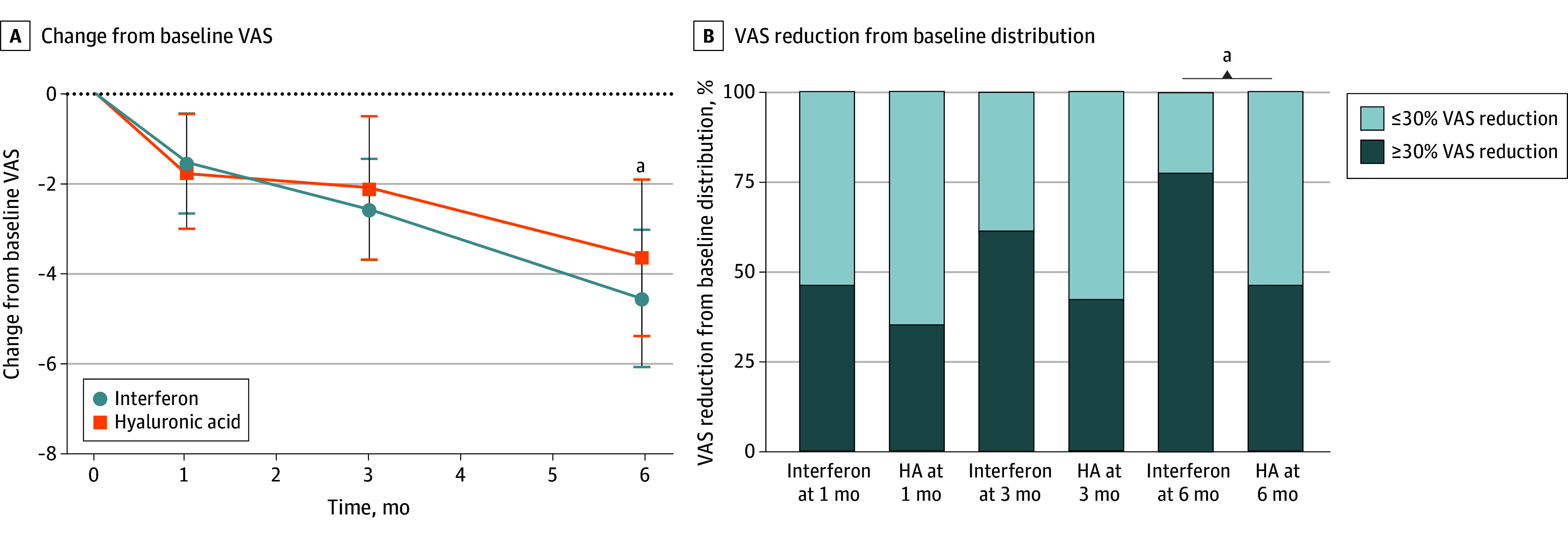
Change From Baseline in Primary Outcome Measures Comparing Interferon With Hyaluronic Acid at Each Visit HA indicates hyaluronic acid; VAS, visual analog scale. Error bars indicate 95% CIs. ^a^*P* < .05.

The primary end point VAS score decreased significantly cvcompared with baseline in both the interferon alpha-2b and HA groups, but did not show a statistically significant difference between 2 groups at month 1 (−0.2; 95% CI, −1.4 to 1.1; *P* = .76) and month 3 (−0.8; 95% CI, −1.9 to 0.3; *P* = .15). However, by month 6, there was a statistically significant difference in the VAS score compared with baseline between both the interferon alpha-2b and HA groups (−1.3; 95% CI, −2.3 to −0.3, *P* = .02). In the first month, only 12 patients in the interferon alpha-2b group (46%) and 9 patients in the HA group (35%) exhibited a 30% or more reduction in pain compared with baseline. However, as time progressed, the effectiveness increased to 20 patients in the interferon alpha-2b group (77%) and 12 patients in the HA group (46%) at month 6, with a significant difference (relative risk [RR], 1.7; 95% CI, 1.1 to 2.7; *P* = .045) (Table 2 and Figure 2). From the current results of VAS score, the effectiveness of interferon therapy appeared to increase with prolonged duration; however, support for longer-term follow-up results would be required.

**Table 2.** Changes in Efficacy Variables From Baseline to 1-Month, 3-Month, and 6-Month Follow-Up in the Interferon Group Compared With the Hyaluronic Acid Group

Outcomes	Patient, mean (SE)		Difference (95% CI)	*P* value
	Interferon alpha-2b (n = 26)	Hyaluronic acid (n = 26)		
**24-hour Voiding frequency**				
1 mo	−2.0 (0.4)	−2.2 (0.4)	0.5 (−4.1 to 5.0)	.84
3 mo	−3.7 (0.6)	−3.1 (0.6)	−0.4 (−5.2 to 4.5)	.88
6 mo	−6.5 (0.7)	−4.4 (0.7)	−1.8 (−6.5 to 2.9)	.44
**Functional bladder capacity, mL**				
1 mo	43.6 (19.9)	26.4 (19.9)	31.8 (−51.7 to 115.3)	.44
3 mo	66.4 (20.8)	33.6 (20.8)	47.3 (−33.9 to 128.4)	.24
6 mo	81.0 (23.5)	40.9 (23.5)	54.5 (−26.8 to 135.9)	.18
**Nocturia episodes**				
1 mo	−0.9 (0.3)	−0.4 (0.3)	−1.5 (−3.8 to 0.7)	.17
3 mo	−1.4 (0.4)	−1.2 (0.4)	−1.2 (−3.5 to 1.1)	.30
6 mo	−1.8 (0.4)	−1.4 (0.4)	−1.5 (−3.7 to 0.8)	.20
**ICSI score**				
1 mo	−2.3 (0.3)	−1.6 (0.3)	−1.6 (−3.0 to −0.3)	.02
3 mo	−3.9 (0.5)	−2.7 (0.5)	−2.2 (−4.2 to −0.2)	.04
6 mo	−5.5 (0.6)	−3.5 (0.6)	−3.0 (−5.3 to −0.7)	.01
**ICPI score**				
1 mo	−2.0 (0.3)	−1.1 (0.3)	−1.5 (−2.4 to 0.8)	.15
3 mo	−3.5 (0.5)	−2.9 (0.5)	−1.1 (−2.8 to 0.6)	.20
6 mo	−5.5 (0.6)	−3.5 (0.6)	−2.5 (−4.5 to −0.4)	.02
**VAS score**				
1 mo	−1.5 (0.4)	−1.7 (0.4)	−0.2 (−1.4 to 1.1)	.76
3 mo	−2.5 (0.4)	−2.1 (0.4)	−0.8 (−1.9 to 0.3)	.15
6 mo	−4.5 (0.5)	−3.6 (0.5)	−1.3 (−2.3 to −0.3)	.02
**≥30% VAS reduction from baseline, No. (%)**				
1 mo	12 (46)	9 (35)	1.3 (0.7 to 2.6)^a^	.57
3 mo	16 (61)	11 (42)	1.5 (0.8 to 2.5)^a^	.27
6 mo	20 (77)	12 (46)	1.7 (1.1 to 2.7)^a^	.05
**GRA responders, No. (%)**				
1 mo	8 (31)	6 (23)	1.3 (0.5 to 3.3)^a^	.76
3 mo	13 (50)	12 (46)	1.1 (0.6 to 1.9)^a^	>.99
6 mo	22 (85)	14 (54)	1.6 (1.1 to 2.3)^a^	.03

Abbreviations: GRA, global response assessment; ICPI, interstitial cystitis problem index; ICSI, interstitial cystitis symptom index; VAS, visual analog scale.

^a^ Relative risk (95% CI).

**Figure 3.**
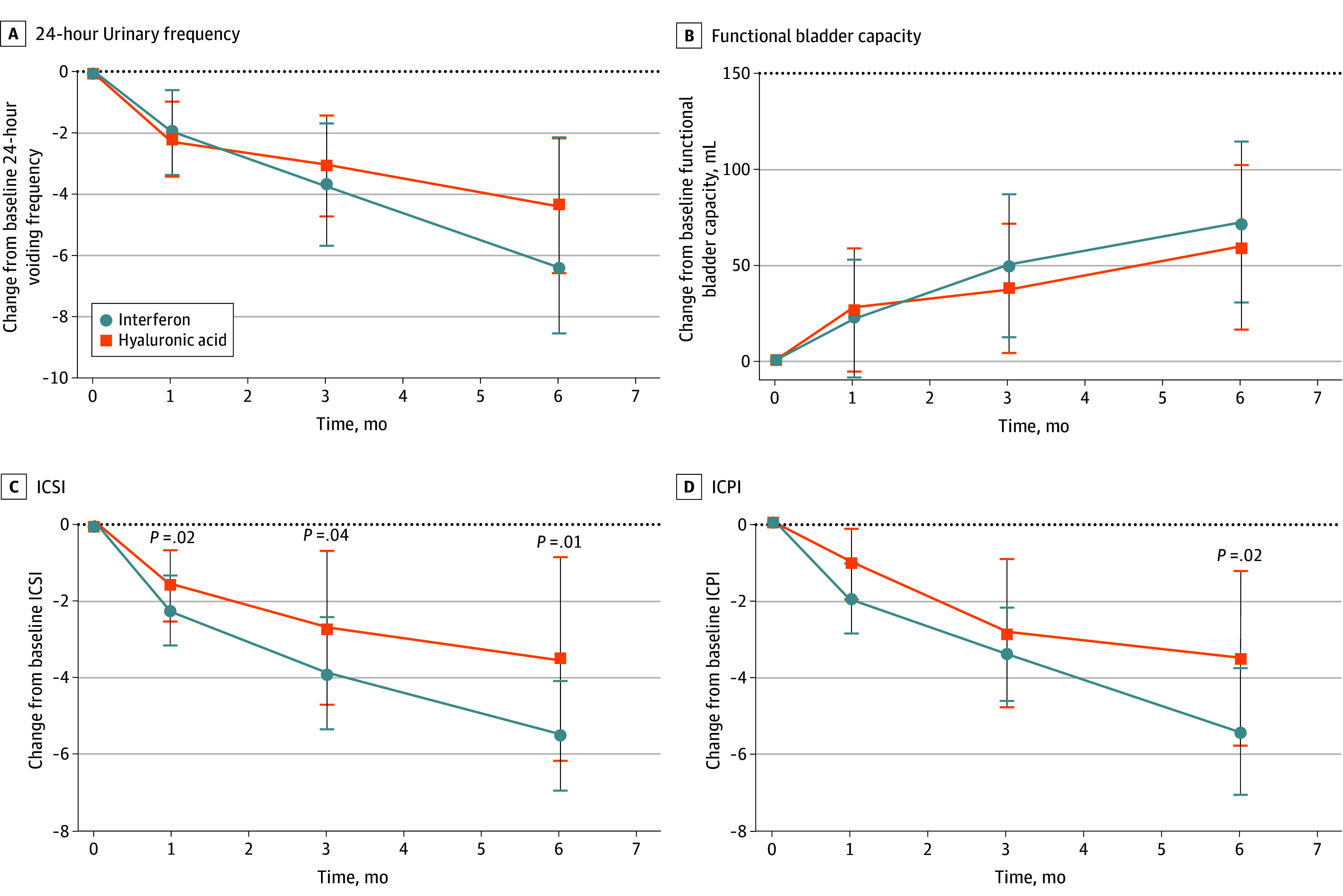
Change From Baseline in Secondary Outcome Measures Comparing Interferon With Hyaluronic Acid ICPI indicates interstitial cystitis problem index; ICSI, interstitial cystitis symptom index. Error bars indicate 95% CIs.

There was no statistically significant difference in secondary end points compared with baseline between the interferon alpha-2b and HA groups at month 6 in 24-hour voiding frequency, functional bladder capacity, and nocturia episodes. However, there was a statistically significant difference in ICSI compared with baseline between the interferon alpha-2b and HA groups, with −1.6 (95% CI, −3.0 to −0.3; *P* = .02) at month 1, −2.2 (95% CI, −4.2 to −0.2; *P* = .04) at month 3, and −3.0 (95% CI, −5.3 to −0.7; *P* = .01) at month 6. Meanwhile, there was a statistically significant difference in ICPI compared with baseline between the 2 groups at month 6, with a difference of −2.5 (95% CI, −4.5 to −0.4; *P* = .021). In the first month, only 8 patients in the interferon alpha-2b group (31%) and 6 patients in HA group (23%) rated GRA as moderately improved or markedly improved (RR, 1.3; 95% CI, 0.5 to 3.3; *P* = .76). However, as time progressed, the number of patients experiencing improvement increased to 13 (50%) in the interferon alpha-2b and 12 (46%) in the HA group at month 3 (RR, 1.1; 95% CI, 0.6 to 1.9; *P* > .99), and it showed a significant difference at month 6, with 22 (85%) in the interferon alpha-2b and 14 (54%) in the HA group showed at least a 2-point improvement in GRA (RR, 1.6; 95% CI, 1.1 to 2.3; *P* = .03). (Table 2, Figure 3, and eFigure in Supplement 2).

### Safety

The study therapy was well tolerated with no serious adverse events. Adverse event rates were similar between groups (eTable in Supplement 2). The most common adverse event reported was bladder irritation, which occurred in 4 patients in the interferon group (15%) and 2 patients in the HA group (8%) (RR, 2.0; 95% CI, 0.4 to 10.0; *P* = .67). Bladder irritation occurred immediately after interferon infusion, and typically self-resolved within 1 week without the need for medication. The second most common complication was a microbiologically documented urinary infection, which occurred in 3 patients in the interferon group (12%) and 2 patients in the HA group (8%) (RR, 1.5; 95% CI, 0.3 to 8.2; *P* >.99). All urinary infections were resolved with appropriate antibiotics.

## Discussion

In this randomized clinical trial, the intravesical instillation of interferon in patients with IC improved patient-reported outcomes (eg, bladder pain, ICSI, and ICPI scores) and objective outcomes (eg, GRA scores) compared with HA. The treatment was also well tolerated.

Intravesical HA instillation is considered as a third-line treatment option for IC.^10^ HA does not appear to be integrated into cell membrane proteoglycans but binds to a number of receptors expressed by urothelial cells.^23^ Although previous results demonstrated a wide range of symptomatic improvement rates, from 30% to 87% after intravesical instillations of HA,^9^ there are still uncertainties regarding the long-term effectiveness of HA therapy. Most retrieved studies are nonrandomized and had scarce numbers.^9,10^

The rationale for exploring intravesical interferon therapy lies in the growing body of evidence suggesting a viral etiology or viral involvement in IC, particularly the potential role of BK virus and JC virus. A previous study^13^ has reported that intravesical cidofovir treatment was effective in decreasing viral loads of JC virus and BK virus, with no observed adverse effects. Another recent study^24^ evaluated the efficacy and safety of certolizumab pegol compared with placebo in female patients with refractory IC, and the results suggested that certolizumab pegol was effective in female patients with moderate to severe refractory IC. In a recent unpublished clinical trial, oral valacyclovir treatment was given to 4 HIC and 24 NHIC patients with EBV infections coexisting in urine sample. This regimen significantly improved VAS scores, and EBV became undetectable in urine samples postvalacyclovir treatment. Based on the results of these pilot studies, it appears that antiviral drug treatment holds promise as a therapeutic option for patients with IC. Our study addresses the efficacy and safety of intravesical interferon instillation therapy in patients with IC. Classifying patients with IC into BK virus positive or BK virus negative groups may prove useful for future research about markers of disease prognosis and the subtypes of IC.

Phenotyping based on specific patient characteristics, including subtype and potential viral involvement, can guide treatment decisions. HIC and NHIC subtypes were differentiated by cystoscopy according to the Japanese Guideline,^25^ and researchers reported they are clinically and pathologically distinct.^26,27^ Thus, conducting a comparative study between the HIC and NHIC subtypes to assess therapy efficacy would be a valuable direction for future research, potentially leading to more personalized and effective therapeutic strategies for patients with IC.

In recent studies, bladder instillations using dimethyl sulfoxide were reported with increased bladder discomfort (8.2%), irritation (10.2%), and pain (30.6%), leading to participants withdrawing from the treatment.^28^ Interestingly, in our study, 4 patients treated with intravesical interferon experienced bladder irritation, but none of them withdrew due to the discomfort during and after the instillations. Bladder irritation typically self-resolved within 1 week without the need for medication. These data showed that majority of patients could be able to tolerate interferon instillations. In fact, our results showed intravesical interferon therapy was more effective in decreasing VAS scores than HA. While there were statistically significant differences in VAS and ICSI scores at specific time points, assessing their clinical relevance was equally crucial. First, the observed improvement in symptoms suggested a substantial alleviation in pain, frequency, and other symptomatic aspects, thereby enhancing patients’ overall quality of life. Second, the introduction of interferon provided a novel therapeutic option particularly for those previously unresponsive to or intolerant of conventional treatments. Moreover, this study represented the first randomized clinical trial to demonstrate the efficacy of antiviral therapy, particularly against the BK virus and JC virus. Compared with previous treatments, this new strategy offered patients a cost-effective option without serious adverse events. Importantly, this study introduced a novel therapeutic mechanism for patients with IC and presented an effective and potentially cost-effective treatment approach.

### Limitations

This study has limitations, including the sample size and follow-up time. Future work should include larger-scale randomized clinical trials with long-term follow-up and the absence of subgroup analyses based on factors such as patient age and severity of symptoms. Additionally, the study’s sample size calculation was based on detecting a difference in the primary outcome. In the future, long-term follow-up and well-designed large-scale randomized clinical trials are needed to assess the lasting effectiveness and safety of the procedure. Additionally, further research may explore the most effective dose and treatment duration of interferon to treat IC.

## Conclusions

This study supports the hypothesis that intravesical interferon may be a potential effective treatment option for patients with IC. This research underscores the potential benefits of antiviral approaches in IC management, which may improve patient care and quality of life. Further prospective multicenter evaluation is necessary to validate these findings.

## Notes

Supplement 1.
Trial Protocol

Supplement 2. **eTable.** Adverse Events Between Treatment Groups **eFigure.** Change From Baseline in Secondary Outcome Measures Comparing Interferon With Hyaluronic Acid

Supplement 3.
Data Sharing Statement
