# Errors in Results and Tables

**DOI:** 10.1001/jamanetworkopen.2024.15703

**Published:** 2024-04-30

In the Original Investigation titled “Intravesical Interferon Therapy vs Hyaluronic Acid for Pain Among Female Individuals With Interstitial Cystitis: a Randomized Clinical Trial,”^1^ published April 8, 2024, there were numerical errors in the results section and Tables. The mean (SD) voiding frequency in 24 hours for the hyaluronic acid group should have read 19.7 (4.5) times. Additionally, in the first paragraph of the Results section, the mean (SD) values for nocturia episodes and functional bladder capacity were swapped. This article has been corrected.^1^
