# Supplementary material for: Intravesical Interferon Therapy vs Hyaluronic Acid for Pain Among Female Individuals With Interstitial Cystitis: A Randomized Clinical Trial
Source: JAMA Netw Open. 2024 Apr 8;7(4):e244880. doi: 10.1001/jamanetworkopen.2024.4880 (PMC11002698; doi:10.1001/jamanetworkopen.2024.4880)
Supplement: Supplement 1. — Trial Protocol [file jamanetwopen-e244880-s001.pdf]

Clinical trial protocol

Title: Effect of Intravesical Interferon therapy in women with Interstitial Cystitis/Bladder Pain Syndrome: A Randomized Clinical Trial.

Contents:

1.0 Introduction
1.1 Rationale
1.2 Study Objectives
2.0 Study Background
2.1 IC/BPS Prevalence and Impact
2.2 Current Treatment Challenges
2.3 Viral Involvement in IC/BPS
2.4 The Role of Interferon
2.5 Hypothesis
3.0 Study Design
3.1 Trial Design
3.2 Study Period
3.3 Ethics and Registration
4.0 Study Participants
4.1 Inclusion Criteria
4.2 Exclusion Criteria
5.0 Randomization and Interventions
5.1 Randomization
5.2 Treatment Groups
6.0 Main Outcomes and Measures
6.1 Primary Endpoint
6.2 Secondary Endpoints
6.3 Safety Monitoring
7.0 Statistical Analysis

7.1 Sample Size Calculation
7.2 Data Analysis
8.0 Discussion
8.1 Clinical Significance
8.2 Anticipated Outcomes
8.3 Limitations and Future Directions
9.0 Data management
10.0 Study schedule
11.0 References

1.0 Introduction

1.1 Rationale

Interstitial Cystitis/Bladder Pain Syndrome (IC/BPS) is a chronic and debilitating condition characterized by urinary symptoms, including increased urinary frequency, urgency, and pelvic discomfort or pain, often exacerbated during bladder filling. It affects millions of individuals worldwide and severely impacts their quality of life. IC/BPS predominantly affects females, with a prevalence ranging from 2% to 17.3% (1), making it a significant public health concern. Despite extensive research, the precise pathogenesis and underlying mechanisms of IC/BPS remain elusive, leading to challenges in both diagnosis and treatment.

Current therapeutic options are limited and often provide only short-term relief. In cases where other treatment modalities fail, patients may resort to invasive surgeries, with a 23% risk of surgical interventions failing to improve their symptoms (2). Thus, there is an urgent need to deepen our understanding of the molecular mechanisms underlying IC/BPS to provide more effective treatments and improve patient care.

1.2 Study Objectives

The primary objective of this study is to assess the efficacy and safety of intravesical interferon instillation as a novel therapeutic approach for women with IC/BPS compared to hyaluronic acid. We aim to determine whether intravesical interferon offers a more effective and long-lasting treatment option for IC/BPS, and whether it can improve the quality of life of affected individuals.

2.0 Study Background

2.1 IC/BPS Prevalence and Impact

IC/BPS imposes a significant burden on patients' daily lives, affecting their emotional, social, and physical well-being. The increased frequency and urgency of urination, along with pain, create constant discomfort and psychological stress. Patients often report difficulty in maintaining employment, social interactions, and sexual relationships due to the condition. This underscores the importance of finding more effective treatments to alleviate symptoms and improve the quality of life of those affected.

2.2 Current Treatment Challenges

While a variety of treatment approaches exist, such as oral medications, bladder instillations, and physical therapy, the efficacy of these treatments varies among individuals. Intravesical hyaluronic acid (HA) instillation is considered a third-line treatment option for IC/BPS and is used to improve the defective bladder glycosaminoglycan layer. However, the effectiveness of HA therapy is still uncertain, and many patients experience relapses after discontinuation (3-5). Additionally, there is a lack of long-term follow-up data on the outcomes of HA therapy.

2.3 Viral Involvement in IC/BPS

Recent research has suggested a possible viral etiology in the development of IC/BPS. Some studies have detected a remarkably high positivity rate of BK polyoma virus (BKPyV) and JCPyV in the urine of IC/BPS patients using single-cell sequencing (6-8). Furthermore, it has been reported that intravesical cidofovir treatment was effective in decreasing viral loads of JCPyV and BKPyV, resulting in symptom reduction.

2.4 The Role of Interferon

Interferon, a group of signaling proteins, plays a crucial role in the innate immune system and is known for its antiviral properties (9, 10). Previous research has suggested that interferon can negatively regulate JCPyV and BKPyV infections. This raises the possibility of interferon being a potential specific drug for the treatment of IC/BPS. The current study is the first to explore the efficacy and safety of interferon intravesical instillation for women with IC/BPS compared to HA.

2.5 Hypothesis

It is hypothesized that intravesical interferon instillation will lead to a significant reduction in pain, as measured by the Visual Analog Scale (VAS) score, and will improve IC/BPS symptoms and patient-reported outcomes compared to hyaluronic acid. The study will aim to test this hypothesis rigorously.

3.0 Study Design

3.1 Trial Design

This study employs a double-masked, randomized, phase II/III clinical trial with a parallel group design. A randomized trial design will help ensure the comparability of the two treatment groups, reducing the potential for selection bias.

3.2 Study Period

The study will be conducted over the course of October 2022 to April 2023. Data analysis and interpretation are anticipated to occur between October and November 2023.

3.3 Ethics and Registration

This study protocol has undergone a rigorous review process and received approval from the Ethics Committee on Biomedical Research (Ethics Committee on Biomedical Research, West China Hospital of Sichuan University, No.2022370). The trial is also registered on ClinicalTrials.gov with the identifier (NCT05912946).

4.0 Study Participants

4.1 Inclusion Criteria

1) Women aged 18–70 years.
2) Diagnosis of IC/BPS, confirmed in the absence of active infection or tumor, following the guidelines provided by the Canadian Urological Association (CUA) and the American Urological Association (AUA) (3, 4).
3) Symptom duration exceeding six months.
4) O’Leary-Sant Interstitial Cystitis Symptom Index (ICSI) and O’Leary-Sant Interstitial Cystitis Problem Index (ICPI) score of 18 or higher.
5) Willingness to undergo bladder perfusion therapy and actively participate in follow-up assessments.

4.2 Exclusion Criteria

1) History of allergy to interferon or hyaluronic acid.
2) Serious heart, lung, liver, kidney, or blood diseases.

3) Pregnancy or lactation.
4) Urinary tract infection within the last two months.
5) History of hepatitis B, hepatitis C, or human immunodeficiency disease.
6) Recent history of bladder hydrodistension, transurethral resection of the bladder, or sacral neuromodulation within the last three months.
7) Treatment with oral medications or intravesical instillation within the last three months.

5.0 Randomization and Interventions

5.1 Randomization

Randomization of patients will be achieved through computer-generated numbers in a 1:1 ratio, with a predefined sequence. A random assignment number will be allocated to each patient and provided to investigators via telephone to maintain blinding.
Blinding was maintained by a numbering system and the use of an investigator to prepare the study medication who did not meet the patients before or after treatment.

5.2 Treatment Groups

1) Interferon Group: Patients will receive intravesical instillation of 1 mL Recombinant human interferon alpha-2b injection (300 international units) mixed with 40 mL sterile saline.
2) Hyaluronic Acid (HA) Group: Patients will receive intravesical instillation of 40 mg/50 mL HA solution.

Both groups will undergo weekly instillations for four weeks, followed by monthly interferon instillations for the subsequent four months. Patients will be instructed to avoid urinating for at least 30 minutes after each instillation to enhance bladder retention.

6.0 Main Outcomes and Measures

6.1 Primary Endpoint

The primary endpoint of this study is the change in the Visual Analog Scale (VAS) score for pain intensity.

The VAS score measures pain intensity on a numerical rating scale ranging from 0 (indicating no pain) to 10 (indicating the worst pain ever). Clinical importance will be defined as a reduction in pain of approximately 30% from baseline.

6.2 Secondary Endpoints

1) 24-hour voiding frequency.

Functional bladder capacity (maximum voiding volume on a 3-day voiding diary).

2) Nocturia episodes.
3) Interstitial Cystitis Symptom Index (ICSI) score.

The O'Leary-Sant instrument is comprised of Symptom Index (score range: 0-20 points), which contains four questions related to urinary and pain symptoms. The score is calculated by summing the points for each item, and a score ≥6 points indicates IC.

4) Interstitial Cystitis Problem Index (ICPI) score.

The O'Leary-Sant instrument is comprised of Problem Index (score range: 0-16 points), which contains four questions related to urinary and pain symptoms. The score is calculated by summing the points for each item, and a score ≥6 points indicates IC.

5) A symmetric seven-point Global Response Assessment (GRA) scale

GRA scores categorized into 7, 6, 5, 4, 3, 2, and 1, indicating markedly worse to markedly improved status. GRA responder is defined as at least a two-point change in GRA.

6.3 Safety Monitoring

Adverse events will be closely monitored from the time of the first infusion through one month after the last instillation, including urinary tract infection, bladder irritation, urinary flow problems, psychiatric symptoms, fever, liver function abnormalities, kidney function abnormalities, and gastrointestinal symptoms. Routine urine analysis, blood chemistry, and blood routine will be conducted at patient visits.

7.0 Statistical Analysis

7.1 Sample Size Calculation

To achieve 90% power with a 2.5% significance level, we have determined that a minimum of 13 patients is

required in each group. Factoring in a 20% dropout rate, the final sample size is set at 17 for each group.

7.2 Data Analysis

All analyses will be performed on an intention-to-treat basis, including all available participant data at each time point, regardless of treatment adherence. Clinical data with continuous variables will be presented as the mean ± standard deviation, while categorical variables will be presented as percentages. Proportions will be compared using the $\chi 2$ test.

Primary and secondary outcome measurements will be analyzed using repeated measures analysis to examine differences between time-based measurements. Statistical significance will be considered when $p < 0.05$. Data analysis will be performed using SPSS version 24.0 for Windows (SPSS Inc., Chicago, IL, USA).

8.0 Discussion

8.1 Clinical Significance

This study aims to address the pressing need for effective treatment options for IC/BPS, a debilitating condition affecting millions of people, predominantly women. By exploring the efficacy of intravesical interferon instillation compared to the current treatment standard, hyaluronic acid, the study seeks to offer a novel therapeutic approach with the potential to alleviate the pain and discomfort associated with IC/BPS and improve the quality of life for affected individuals.

8.2 Anticipated Outcomes

We anticipate that this study will provide valuable insights into the use of interferon as a potential therapeutic option for IC/BPS, offering a ray of hope for patients who have not responded to conventional treatments. The research may also pave the way for antiviral approaches in managing IC/BPS, leading to improved patient care.

8.3 Limitations and Future Directions

The study acknowledges several limitations, such as the need for larger-scale, randomized,

placebo-controlled trials and long-term follow-up. Further research could explore the most effective dose and duration of interferon treatment for IC/BPS.

9.0 Data management

Prof. Deyi Luo from West China Hospital will work with staff of research team to maintain quality assurance for all data. The process will be checked according to Standard Protocol Items: recommendations for interventional trials. All the data will be stored in West China Hospital and are password-protected. All patients' information is only accessible for personnel participating in the study. For statistical analysis, all patients' information will be encoded in order to guarantee personal information and unidentifiable during processing or reporting the result. The number of patients who drop out will be reported.

10.0 Study schedule

Table 1. Schematic view of Standard Protocol Item and study schedule with timing of study visits and content of each visit.

	**Baseline**	**Follow-up**								
		**Week 1**	**Week 2**	**Week 3**	**Week 4**	**Month 2**	**Month 3**	**Month 4**	**Month 5**	**Month 6**
**ENROLMENT**										
Eligibility screen	X									
Informed consent	X									
Allocation	X									
**INTERVENTIONS**										
Interferon group		X	X	X	X	X	X	X	X	
Hyaluronic Acid group		X	X	X	X	X	X	X	X	
**ASSESSMENT**										
24-hour voiding frequency	X		X	X	X	X	X	X	X	X
Functional bladder capacity	X		X	X	X	X	X	X	X	X
Nocturia episodes	X		X	X	X	X	X	X	X	X
ICSI score	X		X	X	X	X	X	X	X	X
ICPI score	X		X	X	X	X	X	X	X	X
VAS score	X		X	X	X	X	X	X	X	X
≥30% VAS reduction			X	X	X	X	X	X	X	X
GRA responders			X	X	X	X	X	X	X	X
**ADVERSE EVENTS MONITORING**										
Interferon group		X	X	X	X	X	X	X	X	X
Hyaluronic Acid group		X	X	X	X	X	X	X	X	X

11.0 References

1. Davis NF, Brady CM, Creagh T. Interstitial cystitis/painful bladder syndrome: epidemiology, pathophysiology and evidence-based treatment options. Eur J Obstet Gynecol Reprod Biol. 2014;175:30-7.
2. Osman NI, Bratt DG, Downey AP, Esperto F, Inman RD, Chapple CR. A Systematic Review of Surgical interventions for the Treatment of Bladder Pain Syndrome/Interstitial Cystitis. Eur Urol Focus. 2021;7(4):877-85.
3. Cox A, Golda N, Nadeau G, Curtis Nickel J, Carr L, Corcos J, et al. CUA guideline: Diagnosis and treatment of interstitial cystitis/bladder pain syndrome. Can Urol Assoc J. 2016;10(5-6):E136-e55.
4. Hanno PM, Erickson D, Moldwin R, Faraday MM. Diagnosis and treatment of interstitial cystitis/bladder pain syndrome: AUA guideline amendment. J Urol. 2015;193(5):1545-53.
5. Wyndaele JJJ, Riedl C, Taneja R, Lovász S, Ueda T, Cervigni M. GAG replenishment therapy for bladder pain syndrome/interstitial cystitis. Neurourol Urodyn. 2019;38(2):535-44.
6. Peng L, Jin X, Li BY, Zeng X, Liao BH, Jin T, et al. Integrating single-cell RNA sequencing with spatial transcriptomics reveals immune landscape for interstitial cystitis. Signal Transduct Target Ther. 2022;7(1):161.
7. Eisen DP, Fraser IR, Sung LM, Finlay M, Bowden S, O'Connell H. Decreased Viral Load and Symptoms of Polyomavirus-Associated Chronic Interstitial Cystitis after Intravesical Cidofovir Treatment. Clinical Infectious Diseases. 2009;48(9):e86-e8.
8. Robles MTS, Cantalupo PG, Duray AM, Freeland M, Murkowski M, van Bokhoven A, et al. Analysis of viruses present in urine from patients with interstitial cystitis. Virus Genes. 2020;56(4):430-8.
9. Fiore T, Martin E, Descamps V, Brochot E, Morel V, Handala L, et al. Indoleamine 2,3-Dioxygenase Is Involved in Interferon Gamma's Anti-BKPyV Activity in Renal Cells. Viruses. 2020;12(8).
10. May D, Bellizzi A, Kassa W, Cipriaso JM, Caocci M, Wollebo HS. IFNα and β Mediated JCPyV Suppression through C/EBPβ-LIP Isoform. Viruses. 2021;13(10).
