# Supplementary material for: Intravesical Interferon Therapy vs Hyaluronic Acid for Pain Among Female Individuals With Interstitial Cystitis: A Randomized Clinical Trial
Source: JAMA Netw Open. 2024 Apr 8;7(4):e244880. doi: 10.1001/jamanetworkopen.2024.4880 (PMC11002698; doi:10.1001/jamanetworkopen.2024.4880)
Supplement: Supplement 2. — eTable. Adverse Events Between Treatment Groups eFigure. Change From Baseline in Secondary Outcome Measures Comparing Interferon With Hyaluronic Acid [file jamanetwopen-e244880-s002.pdf]

Supplemental Online Content

Shen S, Peng L, Zeng X, Zhang J, Shen H, Luo D. Intravesical interferon therapy in interstitial cystitis: a randomized clinical trial. *JAMA Netw Open*. 2024;7(4):e244880. doi:10.1001/jamanetworkopen.2024.4880

eTable. Adverse Events Between Treatment Groups
eFigure. Change From Baseline in Secondary Outcome Measures Comparing Interferon With Hyaluronic Acid

This supplemental material has been provided by the authors to give readers additional information about their work.

© 2024 Shen S et al. *JAMA Network Open.*

eTable. Adverse events between treatment groups.

Adverse events	Interferon alpha-2b (n = 26), No. (%)	Hyaluronic acid (n = 26), No. (%)	RR (95% CI)	P value
Urinary tract infection	3 (12)	2 (8)	1.5 (0.3, 8.2)	1.00
Bladder irritation	4 (15)	2 (8)	2.0 (0.4, 10.0)	0.67
Urinary flow problems	1 (4)	0		1.00
Psychiatric symptoms	0	0		
Fever	0	0		
Liver function abnormalities	0	0		
Kidney function abnormalities	0	0		
Gastrointestinal symptoms	0	0		

© 2024 Shen S et al. *JAMA Network Open.*

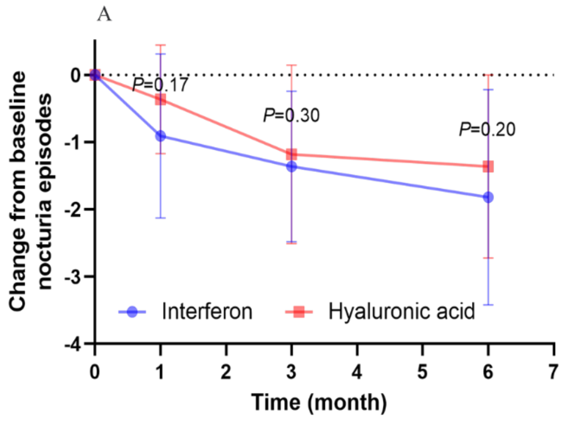

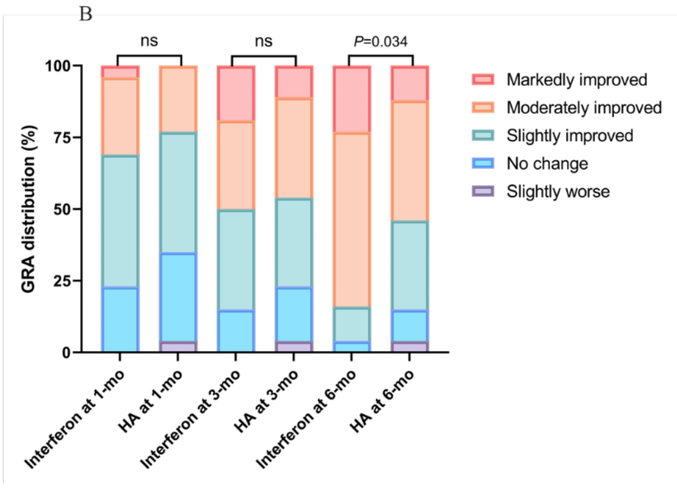

eFigure. Change From Baseline in Secondary Outcome Measures Comparing Interferon With Hyaluronic Acid

(A) Change from baseline in times of nocturia; (B) The distribution of GRA improvement.

GRA = global response assessment.

© 2024 Shen S et al. *JAMA Network Open.*
