# Supplementary material for: Intravesical Interferon Therapy vs Hyaluronic Acid for Pain Among Female Individuals With Interstitial Cystitis: A Randomized Clinical Trial
Source: JAMA Netw Open. 2024 Apr 8;7(4):e244880. doi: 10.1001/jamanetworkopen.2024.4880 (PMC11002698; doi:10.1001/jamanetworkopen.2024.4880)
Supplement: Supplement 3. — Data Sharing Statement [file jamanetwopen-e244880-s003.pdf]

Data Sharing Statement

Data

Data available: Yes
Data types: Deidentified participant data
How to access data: Deidentified data will be made available upon submission of a written request to corresponding author by writing to luodeyi@scu.edu.cn.
When available: With publication

Supporting Documents

Document types: None

Additional Information

Who can access the data: Who can access the data: The data will be made available to researchers for noncommercial purposes.
Types of analyses: The data will be made available to researchers for noncommercial research or educational purposes if similar analyses have not been previously conducted or planned and there are no competing requests.
Mechanisms of data availability: The data will be made available with a with a signed data access agreement and / or a material transfer agreement.
Any additional restrictions: The data may not be made available for commercial purposes or for analyses or research that is already underway or planned, or if there is a competing preceding request for the same set of data and analyses.
